# Around the Hospitals

**Published:** 1892-07-30

**Authors:** 


### Around the Hospitals.

The **Italian Hospital**, Queen's Square, Bloomsbury, does not confine its benefits to those only of Italian nationality, and during the past year 1,675 of the patients treated were English. The institution thus has a large claim on our interest. The hospital has many friends, and great care is exercised in the economical management, consequently the accounts show a balance on the right side. The generosity of artistes is proverbial; from this source the hospital has been greatly benefited during the past year. Since its foundation in 1884 the institution has relieved 1,264 in- and 54,231 out-patients. The treatment is gratuitous, and patients do not require letters of recommendation. The medical staff is principally English. The nursing is by Sisters of S. Vincent de Paul.

The **North London Hospital for Consumption and Diseases of the Chest** has treated 6,370 in-patients and 221,747 out-patients during the thirty years of its existence. The demand on its accommodation becomes every year more pressing, and although a new wing was added in 1880, it is still quite unable to cope with the number of applications for admission, and a new block to contain thirty-five beds is urgently needed. Funds have already been subscribed for this, which warrant the committee commencing the erection of the building, but very much larger sums will be required to defray the cost of the undertaking. A new out-patient's department has been established in Fitzroy Square. Annual subscriptions to the hospital have fallen off during the past year; donations have reached the usual amount, whilst the total income of the institution shows a slight increase on that of previous years. There are samaritan and incurable funds in connection with the hospital.

**St. Mary's Hospital, Paddington**, has passed through a very eventful period of its existence during the past year. An important change in its constitution has been effected, whereby the management, which has hitherto been conducted by the whole body of governors, is now vested in an elective board of management, and in standing committees appointed by this board, the acts of the board and of the standing committees being subject to the control of quarterly and of special boards of governors. The Board of Management is to consist of governors, of whom eight are to be members of the medical staff or lecturers in the medical school. It was felt that change in the direction of management was necessary, and the movement appears already to work well, as the number of attendances at the weekly meetings of the new board greatly exceed those under the old *régime*. St. Mary's, in common with the majority of hospitals, has suffered financially during the past year, and all the interests that can be brought to bear on increasing the income of the institution are necessary to meet the expenditure. Whilst donations and subscriptions alike show a falling off, the hospital has many generous friends to be grateful to. Mr. T. Thistlethwayte has repeated his annual subscription of £300, Mr. J. Hampton, Mr. J. Freeman, and Mr. Alfred de Rothschild have made large donations, and the Extension Fund has largely benefited by Lady Broadbent's Ball and through the munificence of Messrs. Rothschild and Son. There has been an increase in the expenditure as per occupied bed. This increase is due in part to the adoption of a more liberal diet, and to the higher assessment of the hospital in respect to rates and taxes. The number of in-patients and maternity patients has again increased, these numbering 3,922 and 1,069 respectively. One hundred and thirty-three patients have been sent to convalescent homes, and for this branch of the work a special fund exists. The much needed extension of the hospital, which has long been under discussion, has now been satisfactorily arranged. All the necessary property has been acquired, and it is hoped that the Prince and Princess of Wales will lay the foundation stone of the new building in October, which is to be named the "Clarence Wing" in memory of the late Duke. Prince George of Wales has expressed his willingness to accept the office of President of the hospital.

